# The exon-junction complex helicase eIF4A3 holds therapeutic potential in acute myeloid leukemia

**DOI:** 10.1038/s41375-023-02098-2

**Published:** 2023-11-30

**Authors:** Sophia Miliara, Elisabetta Cozzi, Xiangfu Zhong, Isaac Chan, Karl Ekwall, Sören Lehmann, Andreas Lennartsson, Jiri Bartek, Dimitris C. Kanellis

**Affiliations:** 1 Department of Biosciences and Nutrition, Neo, Karolinska Institute, Huddinge, Sweden; 2 Department of Medicine Huddinge, Center for Hematology and Regenerative Medicine, Karolinska Institute, Stockholm, Sweden; 3 Department of Medical Sciences, Hematology Unit, Uppsala University, Uppsala, Sweden; 4 Hematology Centre, Karolinska University Hospital, Stockholm, Sweden; 5 Danish Cancer Institute, Danish Cancer Society, DK-2100 Copenhagen, Denmark; 6 Department of Medical Biochemistry and Biophysics, Science for Life Laboratory, Division of Genome Biology, Karolinska Institutet, S-171 21 Stockholm, Sweden

**Keywords:** Acute myeloid leukaemia, Biochemistry

## To the editor:

Acute myeloid leukemia (AML) is a diverse hematological cancer characterized by uncontrolled proliferation of myeloid blasts or granulocyte myeloid precursors incapable of terminal differentiation. AML is estimated to represent a low percentage of all newly diagnosed cancer cases but shows increasing incidence and death rates with age [1]. While the causal role and prognostic value of key AML driver mutations have been widely explored [2], the effect of aberrant gene expression or post-transcriptional regulation is less studied, yet equally important. For example, it has been found that alternative splicing of DHX34 induces the expression of nonsense-mediated decay (NMD) products that promote AML [3] and that profiling of gene splicing adds prognostic value to AML-related signatures [4]. Exploration of such avenues may offer new therapeutic approaches since currently a substantial fraction of patients either do not respond, or develop resistance to standard-of-care therapy [5].

EIF4A3 (also known as DDX48), the core RNA helicase of the exon junction complex (EJC), is known to regulate RNA polymerase I and II-associated post-transcriptional events [6]. High expression of eIF4A3 is usually associated with disease progression and poor prognosis in various cancer types [6], which makes it an appealing candidate for cancer therapy. Despite the increasing interest in post-transcriptional mechanisms that affect the onset and progression of AML, eIF4A3 and by extension the EJC has never been previously studied in this context. Here, we provide evidence supporting the dependency of AML on eIF4A3 and we show that high eIF4A3 levels correlate with aberrant expression of genes controlling ribosome biogenesis-associated post-transcriptional mechanisms such as rRNA processing and translation. We further demonstrate that genetic depletion or chemical inhibition of eIF4A3 induces leukemic cell death. Our findings reveal a previously undescribed role for eIF4A3 in AML and suggest eIF4A3 inhibition as a potential therapeutic strategy.

**Fig. 1:**
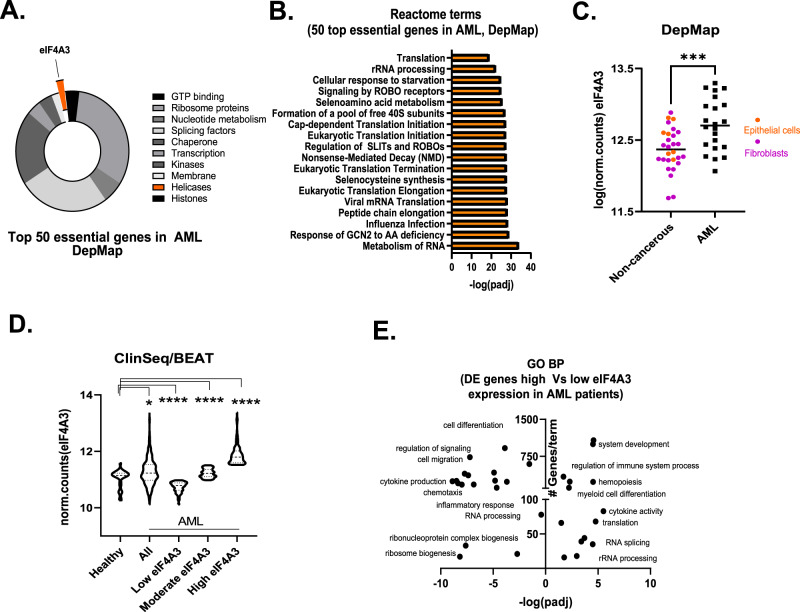
High expression of eIF4A3 in AML correlates with deregulated post-transcriptional events and ribosome biogenesis. **A** Pie chart showing the categorization of the 50 most essential genes in AML based on their molecular function (DepMap). **B** Barplot of the top Reactome pathway enrichment terms for the top 50 most essential genes in AML. **C** EIF4A3 expression analysis in AML and normal-like (non-cancerous) cell lines. RNA-Seq data were extracted from the DepMap repository. *N* = 22 AML and 28 non-cancerous cell lines, ***p = 0.01. **D** EIF4A3 expression in AML and CD34+ bone marrow-derived samples from healthy donors (ClinSeq, BEAT-AML cohorts). AML patients have been sub-grouped based on their eIF4A3 mRNA levels (low: low 25% quartile, high: upper 25% quartile, moderate: second and third quartile, *p < 0.05, ****p < 0.001). **E** Gene ontology biological process (GO-BP) terms enriched in differentially expressed genes between low and high eIF4A3 expressing AML patients.

To explore essential genes in AML, we utilized the CRISPR-based knockout screen data included in the DepMap database [7]. We found that across 18 different AML cell lines, the 50 most essential genes (Supplementary Table S1A) include mainly ribosome proteins (e.g., RPL11, RPS6) and splicing factors (e.g., SNRPE) pointing towards the importance of post-transcriptional mechanisms in AML (Fig. 1A). Indeed, gene ontology analysis of these essential genes showed enrichment in terms covering translation, ribosome biogenesis, splicing, and NMD (Fig. 1B). Notably, such gene list contained an RNA helicase, eIF4A3, that regulates all of the above-mentioned mechanisms and emerges as a candidate therapeutic target in some cancers [6] (Fig. 1A). Importantly, eIF4A3 was the only helicase found in our analysis and showed the highest degree of essentiality among members of the DEAD box family of enzymes (Supplementary Fig. S1A), implicated in the treatment of both solid tumors and blood cancers [8].

To validate such a potentially central role of eIF4A3 in post-transcriptional mechanisms that may propel AML, we then explored the transcriptomic data of DepMap, focusing on the differences between AML cell lines and non-cancerous diploid cells. Firstly, we found that AML cell lines express higher levels of eIF4A3 compared to non-cancerous cells of various lineages (Fig. 1C). This observation was further validated using clinically relevant CD34+ cells from bone marrow-derived samples (two different cohorts: ClinSeq, BEAT-AML [9, 10]) (Fig. 1D, all versus healthy). Given the commonly occurring inter-tumoral heterogeneity of many genes’ expression, we next assessed the degree of eIF4A3 variability, allowing us to subdivide the AML cases into three subsets: those expressing low (the bottom 25%), medium (26–75%) or high (top 25%) levels, designated Low, Moderate and High, respectively (Fig. 1D). While the Low subset featured eIF4A3 levels even beyond that seen in healthy controls, both the Moderate and particularly the High subsets showed a robust overexpression of eIF4A3 (Fig. 1D). The widely variable expression of eIF4A3 was also validated with qPCR in RNA samples from a small panel of AML patients (ClinSeq) (Supplementary Fig. 1B).

To explore cellular pathways related to eIF4A3 expression, we then performed a differential expression (DE) analysis and found that 1928 genes were up- and 1531 downregulated (Supplementary Table S1B, C) in the AML subset expressing Low eIF4A3 levels compared with High-expressors (identified in Fig. 1D). Such differentially expressed genes showed enrichment in Gene Ontology (GO) terms referring to ribosome biogenesis, translation, and splicing all known to be affected by eIF4A3 (Fig. 1E) [6, 11]. Subsequently, we extended our DE analysis to AML patients versus healthy donors and AML versus non-cancerous cell lines from DepMap (Supplementary Fig. S1C, D and Supplementary Table S1D, E). We found 1871 commonly upregulated and 1214 downregulated genes (Supplementary Table S1F, G) that were then subjected to GO analysis. Again, the upregulated genes belonged to, among other terms, ‘rRNA metabolism’ and ‘translation’ (Supplementary Table S1H). Altogether, these results suggest that post-transcriptional mechanisms such as rRNA processing might provide new targets in AML and be explored in future personalized patient profiling to determine sensitivities to relevant pathway inhibitors, possibly including inhibition of eIF4A3 itself.

**Fig. 2:**
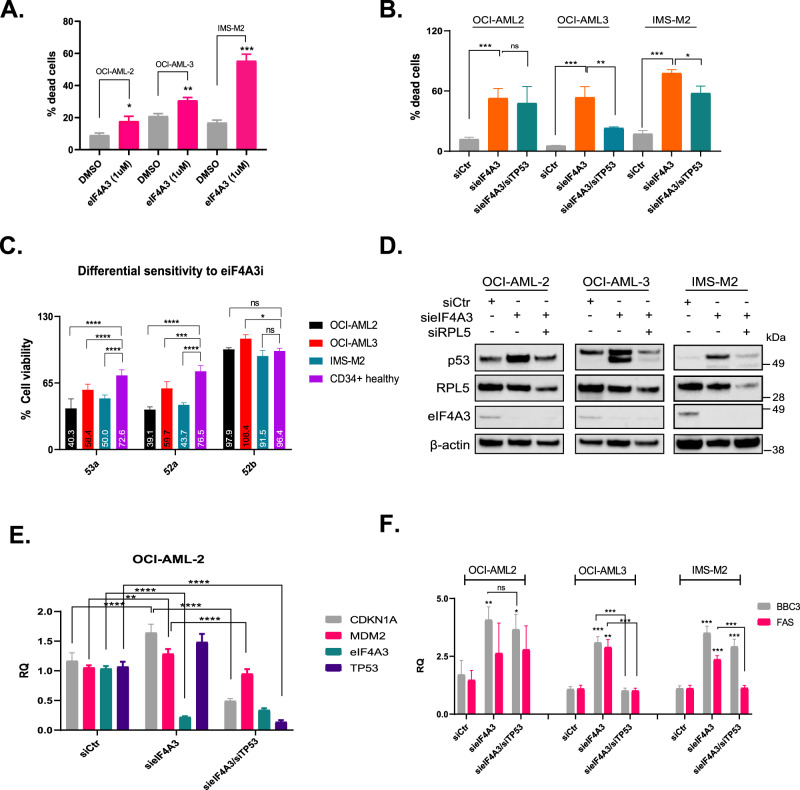
The eIF4A3/IRBC/p53 axis is essential for AML cell survival. **A** Percentage of OCI-AML-2, OCI-AML-3, or IMS-M2 dead cells in cells treated with the eIF4A3 chemical inhibitor (53a) [13] for 72 h. Data shown as mean ± SD, *n* = 3 biological replicates, ***p = 0.01, **p < 0.01, *p < 0.05. **B** Percentage of dead cells following RNAi-mediated silencing of *eIF4A3* for 72 h +/- RNAi-mediate depletion of *TP53*. Data shown as mean ± SD, *n* = 3 biological replicates, ***p = 0.01, **p < 0.01, *p < 0.05, ns non-significant. **C** Percentage of viable cells following treatment of AML cells or CD34+ bone marrow myeloid cells derived from a healthy donor. Two active eIF4A3 inhibitors (53a, 52a) and one inactive stereoisomer (52b) [13] were used at 4 μM for 48 h. Data shown as mean ± SD, *n* = 3 biological replicates, ****p < 0.001, ***p = 0.01, *p < 0.05, ns non-significant. **D** Immunoblotting showing p53, RPL5 and eIF4A3 levels following knock-down of *eIF4A3* +/- *RPL5*. qRT-PCR analysis of *CDKN1A*, *MDM2*, *eIF4A3*, and *TP53* (**E**) or *BBC3* and *FAS* (**F**) mRNA levels in cells depleted of *eIF4A3* +/- *TP53*. In the absence of designating lines (**F**), the asterisks show the comparison to siCtr. Data shown as mean ± SD, *n* = 3 biological replicates, ****p < 0.001, ***p = 0.01, **p < 0.01, *p < 0.05, ns non-significant.

Indeed, the dependency of AML cells on eIF4A3 suggests that the latter might serve as a therapeutic target. In support of this notion, AML cell lines are more sensitive to CRISPR-mediated eIF4A3 depletion compared to non-cancerous cell lines (DepMap, Supplementary Fig. S2A). Moreover, treatment with an eIF4A3 chemical inhibitor (eIF4A3i, with over 100-fold specificity compared to other eIF4A members or helicases [12, 13]) induced cell death in three different p53^wt^-carrying AML cell line models (Fig. 2A), a phenotype that could be recapitulated when *eIF4A3* was silenced with siRNA (Fig. 2B). Furthermore, healthy donor-originated CD34+ bone marrow cells (Fig. 2C and Supplementary Fig. S2B, C) as well as peripheral blood mononuclear cells (Supplementary Fig. S2D) were less sensitive to eIF4A3 chemical inhibition compared to AML cells further pointing towards a possible therapeutic window.

As p53 was reported to (partially) mediate the effect of eIF4A3 depletion on cell survival [6], we addressed this scenario in AML. Fig. 2B shows that concomitant silencing of *eIF4A3* and *TP53* reversed partially the effect of *eIF4A3* depletion on cell death in two out of three cell lines tested (no response of p53 in OCI-AML2). P53 is the central molecule of the checkpoint induced by impaired ribosome biogenesis (IRBC) [14] that is, a mechanism that is triggered also by *eIF4A3* silencing [6]. Focusing on AML, we showed that siRNA-mediated *eIF4A3* knock-down induces p53 in an IRBC-mediated fashion since concomitant silencing of *RPL5* (a component of the 5S-RNP complex that regulates the IRBC [14]) rescued the effect of *eIF4A3* KD on p53 (Fig. 2D).

*EIF4A3* knock-down led to the activation of p53, shown by the upregulation of its direct targets *CDKN1A* and *MDM2*, an effect that was reversed when *TP53* was simultaneously silenced (Fig. 2E, Supplementary Fig. S2E, F). The activation of the IRBC-p53 axis could explain the cytotoxic effect of eIF4A3 inhibition, at least in p53 proficient cells. Indeed, when we investigated the expression levels of two typical p53 apoptotic targets, *BBC3* (PUMA) and *FAS*, we found that *eIF4A3* silencing induced their expression and this was reversed upon simultaneous silencing of *TP53* (Fig. 2F). Of note, sieIF4A3-mediated *BBC3* upregulation was not dependent on p53 in OCI-AML2 cells (Fig. 2F). This shows that *eIF4A3* silencing can induce apoptotic genes both in a p53-dependent and -independent manner in agreement with previous findings [6] and helps explain the differential sensitivity of AML cell lines to p53-mediated cell death (shown in Fig. 2B).

In conclusion, our data suggest that AML cells are dependent on eIF4A3 and feature aberrant post-transcriptional gene regulation (affecting ribosome biogenesis and other processes), a vulnerability that could be exploited in AML therapy. To this end, we show that eIF4A3 silencing triggers the IRBC and leads to p53 activation and cell death. Our data show also that siRNA-mediated *eIF4A3* depletion drives cell death even in a p53-independent manner, thereby further expanding the therapeutic potential of eIF4A3 inhibition in AML with mutant p53. We hope that our present data may inspire further preclinical and clinical work, including assessment of in vivo feasibility, and search for predictive biomarkers, to identify AML patients who might most benefit from possible therapeutic targeting of eIF4A3 in the future.

### Supplementary information

Supplementary Table S1

Supplementary Information

## Data Availability

All data generated or analyzed during this study are included in this published article and its supplementary information files. The RNA-Seq data were extracted from either the DepMap consortium or the ClinSeq-AML and BEAT-AML cohorts. Any requests for access to ClinSeq-AML data should be made to SL.
