# Supplementary material for: The exon-junction complex helicase eIF4A3 holds therapeutic potential in acute myeloid leukemia
Source: Leukemia. 2023 Nov 30;38(3):663–6. doi: 10.1038/s41375-023-02098-2 (PMC10912025; doi:10.1038/s41375-023-02098-2)

**Methods**

Cell culture and treatments

WT p53 carrying AML cell lines, OCI-AML2, OCI-AML3, and IMS-M2, were grown in RPMI-1640 (Gibco, Paisley, UK) supplemented with 10% Fetal bovine Serum (FBS) (vol/vol) (Gibco, Paisley, UK). Human PBMC (CC-2702) and bone marrow CD34+ cells (2M-101C) were purchased by Lonza and were grown in StemSpam SFEM (Stemcell technologies, 09600) supplemented with StemSpan CC100 (Stemcell technologies, 02690). eIF4A3 small chemical inhibitor (52a, 53a or their inactive stereoisomer 52b) was added in a final concentration of 1μM or otherwise stated with DMSO as a vehicle. These are ATP-competitive inhibitors with high specificity (> 100 fold) for eIF4A3 compared to other members of the eIF4A family or other helicases such as DHX29 and BRR2[1–4]. They were initially designed to block the nonsense-mediated decay function of eIF4A3 but have been shown to inhibit the RNA unwinding activity of eIF4A3 in general, expanding their potency against other activities of eIF4A3 such as the one in ribosome biogenesis that our group has recently described[1]. Cell survival analysis was performed with the XTT assay (Thermo Fischer Scientific, X12223) following the manufacturer's instructions.

Gene silencing

*EIF4A3* (L-020762), *TP53* (L-003329), and *RPL5* (L-013611) knock-down were achieved with SMARTpool ON-TARGET oligonucleotides (Horizon Discovery). Non-targeting siRNA (D-001810) was used as a control. Cells were transfected using the NEON electroporation system (Thermo Scientific) according to the manufacturer's instructions. The final concentration of siRNA was 20nM.

Quantitative real-time PCR

Following RNA extraction with PureLink™ RNA Mini Kit (Thermo Fisher Scientific (12183025), quantitative RT-PCR was conducted with TaqMan™ RNA-to-CT™ 1-Step Kit (Thermo Fisher Scientific, 4392938) in a StepOnePlus™ Real-Time PCR System (Applied Biosystems). Taqman probes used (Thermo Fisher Scientific): EIF4A3: Hs01556773_m1, MDM2: Hs02970282_cn, CDKN1A: Hs00355782_m1, TP53 Hs01034249_m1, BBC3 Hs00248075_m1 and BBC3, FAS Hs00236330_m1, beta-actin Hs01060665_g1, GAPDH Hs02786624_g1.

Western blot

Following chemical administration or transfection, subconfluent cells were lysed in radioimmunoprecipitation assay (RIPA) buffer supplemented with a cocktail of protease and phosphatase inhibitors (Thermo Fisher Scientific, 78444) and sonicated for five cycles of 30-s on and 15-s off, in a Bioruptor (Diagenode). Following lysate clearance with centrifugation for 10 min at 13,000 rpm and 4°C, protein quantification was performed with the DC Protein Assay Kit II (Bio-Rad, 5000112). Cell lysate (10 μg) was boiled in Laemmli sample buffer for 5 min at 95°C, loaded onto SDS–polyacrylamide gel electrophoresis gels and transferred onto nitrocellulose or polyvinylidene difluoride membranes. The chemiluminescence signal was detected using SuperSignal West Dura (Thermo Fisher Scientific, 34076). Images were acquired with an Amersham Imager 600 scanner. The antibodies used are as follows: mouse monoclonal eIF4A3 (Santa Cruz Biotechnology, sc-365549), rabbit polyclonal RPL5 (uL18) (Abcam, ab86863), mouse monoclonal p53 (1:1000; Abcam, ab1101) and mouse monoclonal beta-actin (Abcam, ab6276). All primary antibodies were used at a working dilution of 1:500. Secondary antibodies (mouse horseradish peroxidase HRP; Sigma-Aldrich, A9044 and rabbit HRP, Sigma-Aldrich, A6154) were used in dilution 1:10,000.

Flow cytometry analysis of apoptosis

The effect of *eIF4A3* and *TP53* knock-down or of the EIF4A3 chemical inhibitor on apoptosis of the AML cells was determined by Annexin V-FITC/PI staining or PI staining respectively according to

standard protocols. Samples were analyzed by flow cytometry on a BD FACS Aria II (BD Biosciences) and results were analyzed using FlowJo software (Tree Star Inc., Ashland, OR).

Data mining, statistics, and reproducibility

Cell line-associated eIF4A3 dependency and expression data were retrieved from DepMap (v.23Q2). AML human patient and healthy donor RNA-Seq raw counts and clinical metadata were retrieved from the ClinSeq[5] or the BEAT-AML cohort[6] and were processed using the statistical languare R. Raw RNA-seq count normalization and differential expression analysis was done with the DESEq2 package[7] in R. ClinSeq RNA-seq data (AML and healthy bone marrow samples), were processed by the nf-core rnaseq pipeline (v1.4.2)[8]. More in detail, we used FastQC and MultiQC for quality control; Trim Galore! for adapter trimming; STAR for mapping reads to the human reference genome GRCh37 and featureCounts gene level quantification. The human reference genome annotation GENCODEv39 (gencode.v39lift37.annotation.gtf) was used for gene expression quantification. GO pathway analysis was performed with gprofiler[9] and the calculation of z scores with GO[10] in R. Figures were produced either in R or with GraphPad Prism® (v.9.4.1). Exclusion criteria: For DepMap we exluded AML samples with OncotreeSubtype: 'Acute Monoblastic/Monocytic Leukemia', 'Acute Megakaryoblastic Leukemia','Myeloid Leukemia Associated with Down Syndrome', 'Spherocytosis','AML with Maturation','AML with inv(3)(q21.3q26.2) or t(3;3)(q21.3;q26.2); GATA2, MECOM' and 'Acute Myelomonocytic Leukemia' (N = 22 AML and 28 non cancerous cell lines were used for the differential expression analysis). For clinical data, we focused our analysis on acute myeloid leukemia patients (with BM-derived CD34+ samples) that were not previously or concomitantly presented with other comorbidities. For the ClinSeq cohort we excluded samples with subtype ‘APL, FAB M3’ and used only samples collected from bone marrow. For the BEAT-AML cohort we used healthy donor samples originated from ‘Healthy pooled CD34+’ and AML patients from the group: ‘Initial Acute Leukemia Diagnosis’ and type: ‘Bone marrow aspirate’. Patients with specificDxAtInclusion criteria: 'Acute erythroid leukaemia', 'Acute megakaryoblastic leukaemia','Acute monoblastic and monocytic leukaemia', 'Acute myelomonocytic leukaemia', 'Acute promyelocytic leukaemia with t(15;17)(q22;q12); PML-RARA', 'Acute undifferentiated leukaemia', 'Atypical chronic myeloid leukaemia, BCR-ABL1 negative', 'Blastic plasmacytoid dendritic cell neoplasm', 'Chronic myelomonocytic leukaemia', 'Essential thrombocythaemia', 'Mastocytosis', 'Mixed phenotype acute leukaemia, B/myeloid, NOS', 'Mixed phenotype acute leukaemia, T/myeloid, NOS', 'Myelodysplastic syndrome associated with isolated del(5q)', 'Myelodysplastic syndrome, unclassifiable', 'Myelodysplastic/myeloproliferative neoplasm, unclassifiable', 'Myeloid leukaemia associated with Down syndrome', 'Myeloid sarcoma', 'Plasma cell myeloma', 'Primary myelofibrosis', 'Refractory anaemia with excess blasts', 'Refractory cytopenia with multilineage dysplasia', 'Secondary myelofibrosis' and 'Unknown' were excluded from our analysis (N = 23 healthy donors and 476 AML patients).

References


1 Kanellis DC, Espinoza JA, Zisi A, Sakkas E, Bartkova J, Katsori AM *et al.* The exon-junction complex helicase eIF4A3 controls cell fate via coordinated regulation of ribosome biogenesis and translational output. *Sci Adv* 2021; **7**: 1–19.

2 Ito M, Tanaka T, Cary DR, Iwatani-Yoshihara M, Kamada Y, Kawamoto T *et al.* Discovery of Novel 1,4-Diacylpiperazines as Selective and Cell-Active eIF4A3 Inhibitors. *J Med Chem* 2017; **60**: 3335–3351.

3 Iwatani-Yoshihara M, Ito M, Ishibashi Y, Oki H, Tanaka T, Morishita D *et al.* Discovery and

Characterization of a Eukaryotic Initiation Factor 4A-3-Selective Inhibitor That Suppresses Nonsense-Mediated mRNA Decay. *ACS Chem Biol* 2017; **12**: 1760–1768.

4 Ito M, Iwatani M, Kamada Y, Sogabe S, Nakao S, Tanaka T *et al.* Discovery of selective ATP-competitive eIF4A3 inhibitors. *Bioorganic Med Chem* 2017; **25**: 2200–2209.

5 Wang M, Lindberg J, Klevebring D, Nilsson C, Mer AS, Rantalainen M *et al.* Validation of risk stratification models in acute myeloid leukemia using sequencing-based molecular profiling. *Leukemia* 2017; **31**: 2029–2036.

6 Tyner JW, Tognon CE, Bottomly D, Wilmot B, Kurtz SE, Savage SL *et al.* Functional genomic landscape of acute myeloid leukaemia. *Nature* 2018; **562**: 526–531.

7 Love MI, Huber W, Anders S. Moderated estimation of fold change and dispersion for RNA-seq data with DESeq2. *Genome Biol* 2014; **15**: 1–21.

8 Philip A. Ewels, Alexander Peltzer, Sven Fillinger, Harshil Patel, Johannes Alneberg, Andreas Wilm, Maxime Ulysse Garcia PDT& SN. The nf-core framework for community-curated bioinformatics pipelines. *Nat Biotechnol* 2020; **38**: 271.

9 Raudvere U, Kolberg L, Kuzmin I, Arak T, Adler P, Peterson H *et al.* G:Profiler: A web server for functional enrichment analysis and conversions of gene lists (2019 update). *Nucleic Acids Res* 2019; **47**: W191–W198.

10 Walter W, Sánchez-Cabo F, Ricote M. GOplot: an R package for visually combining expression data with functional analysis. *Bioinformatics* 2015; **31**: 2912–4.

**Supplementary Data**

**Supplementary Figure legends**

**Supplementary Fig. S1. High expression of eIF4A3 in AML correlates with deregulated post-transcriptional events and ribosome biogenesis. A** Comparative analysis of the DDX helicase family essentiality in AML (DepMap). **B** eIF4A3 mRNA levels in 9 different AML patients (ClinSeq) and CD34+ cells from a healthy donor, * $p < 0.05$,** $p < 0.01$. **C** Principal component analysis of normalized (DESEq2)[1] RNA-seq data of AML and non-cancer cell lines (DepMap). **D** Dimension reduction (UMAP) plots for normalized RNA-Seq data from AML patients and healthy donors (ClinSeq and BEAT-AML cohorts).

**Supplementary Fig. S2. The eIF4A3/IRBC/p53 axis is essential for AML cell survival.**

**A** Dependency score of AML versus noncancerous cell lines from DepMap. Highlighted in orange are the noncancerous cell lines of myeloid origin, * $p < 0.05$. **B, C** Comparative analysis of IC50 values for the eIF4A3 inhibitor (53a) (B) or 52a (C) in three different AML cell lines and CD34+ bone marrow-derived myeloid cells. The analysis was performed in duplicates with a total sample size of N = 8. IC50 was calculated using nonlinear data fitting (inhibitors versus response-variable slope, four parameters log(inhibitor) vs. normalized response -- Variable slope) in GraphPad Prism® (v.9.4.). **D** Comparative analysis of IC50 values for the eIF4A3 inhibitor (53a) in three different AML cell lines and human peripheral blood mononuclear cells (PBMC). The analysis was performed in duplicates with a total sample size of N = 8. IC50 was calculated using nonlinear data fitting (inhibitors versus response-variable slope, four parameters) in GraphPad Prism® (v.9.4.). **E,F** qRT-PCR analysis of *CDKN1A*, *MDM2*, *eIF4A3,* and *TP53* mRNA levels in OCI-AML3 (E) or IMS-M2 (F) cells depleted of eIF4A3 +/- TP53 KD. Data are shown as mean ± SD, n = 3 biological replicates, ** $p < 0.01$, **** $p < 0.001$.

**Supplementary Table S1**

**A.** Top 50 essential genes in AML (DepMap v.22Q4)

**B.** Differentially expressed (DE) genes in high Vs low eIF4A3 expressing AML patient samples (CLinSeq & BEAT-AML, padj < 0.05)

**C**. GO BP terms of common differentially expressed genes from (B) (generated with ClueGO in Cytoscape)

**D**. DE genes in AML vs. non-cancerous cell lines (DepMap, padj < 0.05)

**E**. DE genes in AML Vs CD34+ BM samples from healthy donors  (ClinSeq + BEAT-AML cohorts, padj < 0.05)

**F**. Common upregulated DE genes from (D) and (E)

**G**. Common downregulated DE genes from (D) and (E)

**H.** GO BP terms of common DE genes in AML vs. healthy donors (ClinSeq/BEAT) or non-cancerous cell lines (DepMap)

References

1 Love MI, Huber W, Anders S. Moderated estimation of fold change and dispersion for RNA-seq data with DESeq2. *Genome Biol* 2014; **15**: 1–21.

# Supplementary Fig. S1

A.

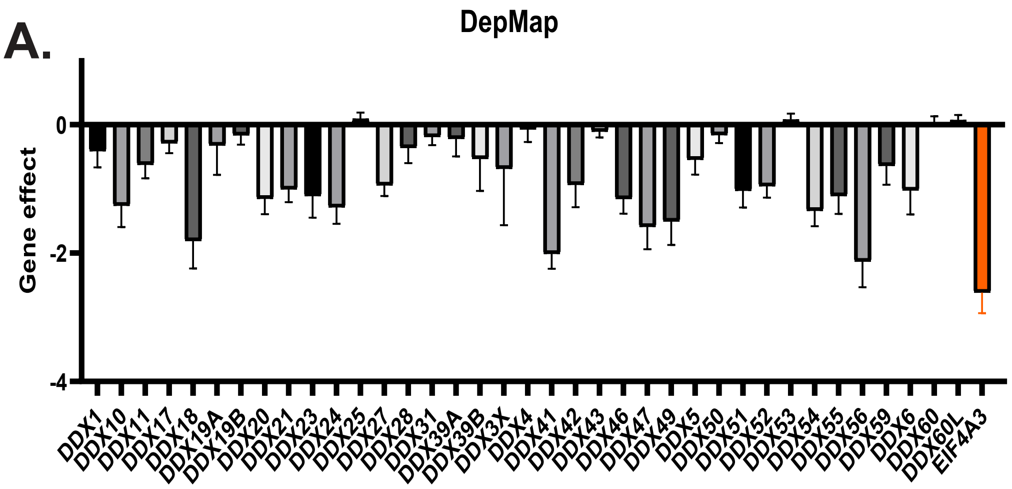


B.

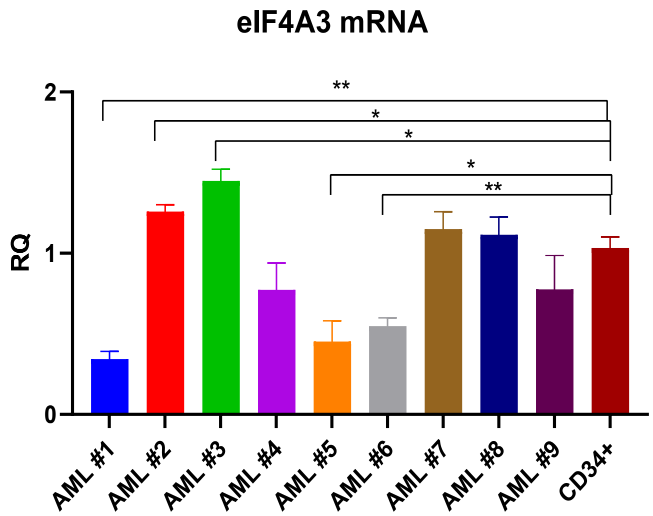


C.

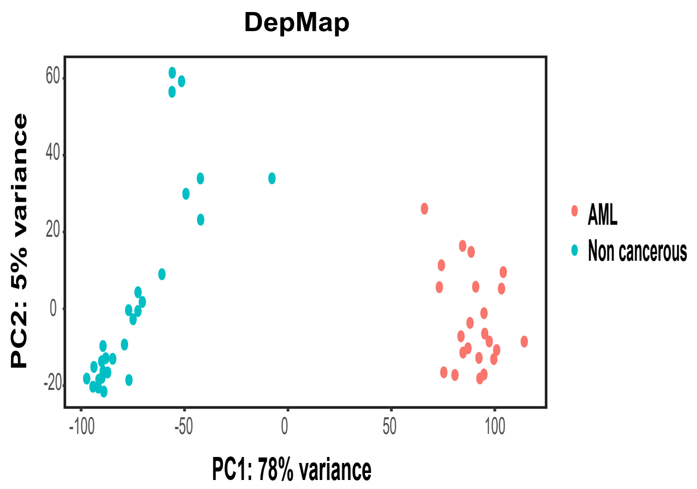


D.

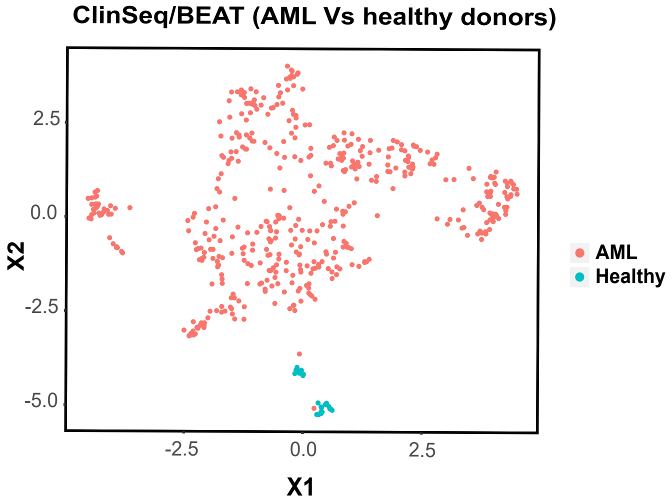

# Supplementary Fig. S2

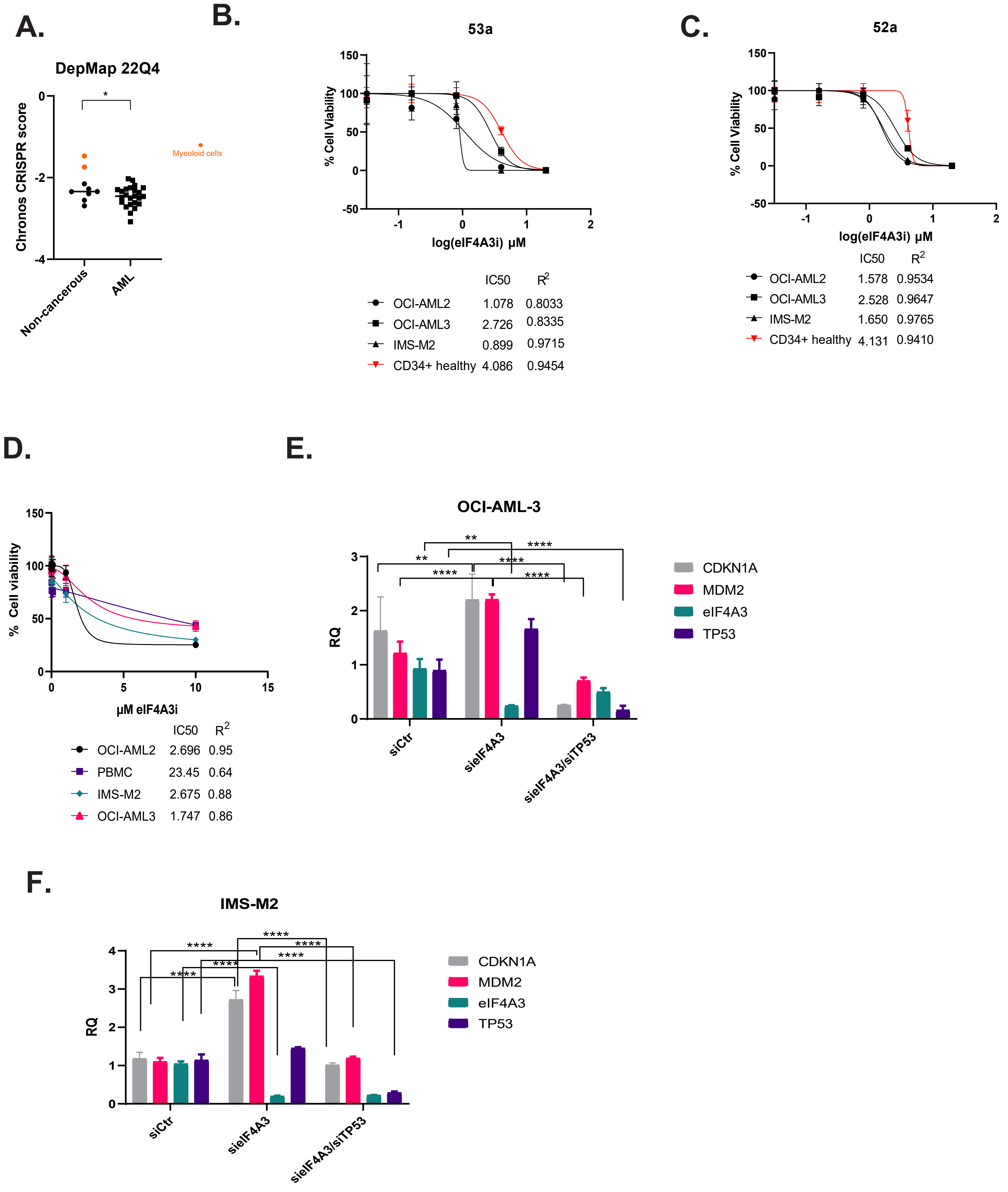

# Uncropped WB images

Figure 2D

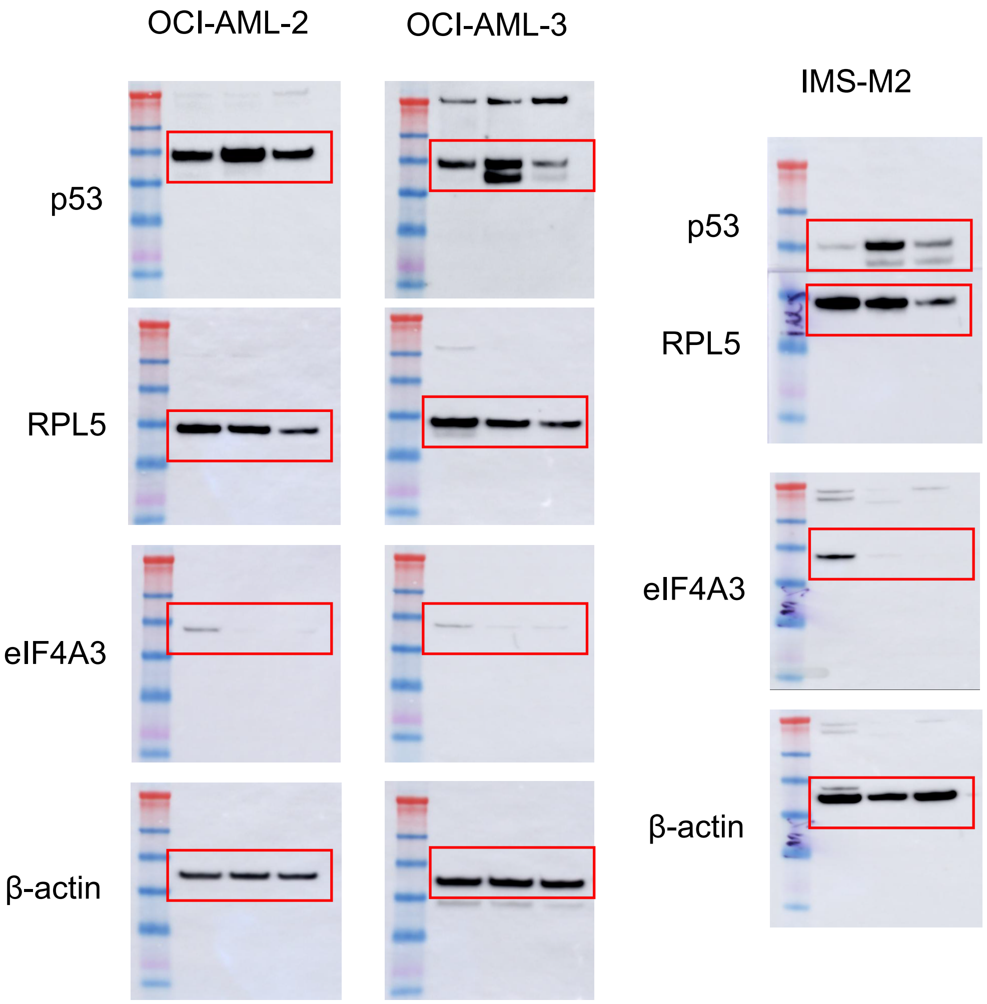

Supplement: Supplementary file 2 — Supplementary Information [file 41375_2023_2098_MOESM2_ESM.pdf]
